# Additional lateral plate fixation has no effect to prevent cage subsidence in oblique lumbar interbody fusion

**DOI:** 10.1186/s13018-021-02725-7

**Published:** 2021-10-11

**Authors:** Tenghui Ge, Jintao Ao, Guanqing Li, Zhao Lang, Yuqing Sun

**Affiliations:** Department of Spine Surgery, Peking University Fourth School of Clinical Medicine, Beijing Jishuitan Hospital, No. 31, Xinjiekou East Street, Xicheng District, Beijing, 100035 People’s Republic of China

**Keywords:** Oblique lumbar interbody fusion, Lumbar degenerative disease, Lateral plate fixation, Cage subsidence, Revision surgery

## Abstract

**Background:**

For lumbar degenerative diseases, cage subsidence is a serious complication and can result in the failure of indirect decompression in the oblique lumbar interbody fusion (OLIF) procedure. Whether additional lateral plate fixation was effective to improve clinical outcomes and prevent cage subsidence was still unknown. This study aimed to compare the incidence and degree of cage subsidence between stand-alone oblique lumbar interbody fusion (SA-OLIF) and OLIF combined with lateral plate fixation (OLIF + LP) for the treatment of lumbar degenerative diseases and to evaluate the effect of the lateral plate fixation.

**Methods:**

This was a retrospective comparative study. 20 patients with 21 levels underwent SA-OLIF and 21 patients with 26 levels underwent OLIF + LP. We compared clinical and radiographic outcomes between two groups. Clinical evaluation included Visual Analog Scale (VAS) for back pain and leg pain, Japanese Orthopaedic Association (JOA) scores and Oswestry Disability Index (ODI). Radiographical evaluation included disc height (DH), segmental lordosis angle (SL), and subsidence rate on standing lateral radiographs. Cage subsidence was classified using Marchi’s criteria.

**Results:**

The mean follow-up duration was 6.3 ± 2.4 months. There were no significant differences among perioperative data (operation time, estimated intraoperative blood loss, and complication), clinical outcome (VAS, ODI, and JOA) and radiological outcome (SH and SL). The subsidence rate was 19.0% (4/21) in SA-OLIF group and 19.2% (5/26) in OLIF + LP group. 81.0% in SA-OLIF group and 80.8% in OLIF + LP group had Grade 0 subsidence, 14.3% in SA-OLIF group and 15.4% in OLIF + LP group had Grade I subsidence, and 4.8% in SA-OLIF group and 3.8% in OLIF + LP group had Grade II subsidence (*P* = 0.984). One patient with severe cage subsidence and lateral plate migration underwent revision surgery.

**Conclusions:**

The additional lateral plate fixation does not appear to be more effective to prevent cage subsidence in the oblique lumbar interbody fusion, compared with stand-alone technique. If severe cage subsidence occurs, it may result in lateral plate migration in OLIF combined with lateral plate fixation.

## Background

Degenerative lumbar disc diseases increase in frequency and severity with age, causing chronic back pain, neurogenic claudication, radiculopathy [1]. Once the conservative strategies prove ineffective, spinal fusion procedures may be used for neural decompression and segmental stabilization through a variety of approaches [1, 2]. Oblique lumbar interbody fusion (OLIF) is a new surgical interbody fusion procedure for degenerative lumbar diseases including discogenic low back pain, lumbar instability, mild lumbar spondylolisthesis, mild‐to‐moderate spinal stenosis, and lumbar degenerative scoliosis [3].

OLIF is one of the minimally invasive techniques using retroperitoneal approach into the disc space between the abdominal aorta and the psoas major muscle [1, 3]. In the OLIF procedure, placement of the large interbody cage in the intervertebral disc space can reduce the bulging of the disc and lengthen the hypertrophic ligamentum flavum by increase disc height to achieve indirect neural decompression [4]. Apart from the clinical benefits of indirect neural decompression, OLIF has the ability to correct deformity and restore segmental alignment with a large interbody cage using ligamentotaxis [5]. Sand-alone OLIF has the advantages of the shorter surgery times, less bleeding, fewer complications, and lower cost than OLIF combined with various supplemental fixation [6, 7]. However, these advantages are limited by postoperative cage subsidence, resulting in the failure of deformity correction and neural decompression [8].

Biomechanical studies have shown that OLIF combined with supplemental fixation, such as pedicle screws and lateral plates, can increase the stability of an interbody fusion construct compared with stand-alone OLIF [9, 10]. The PIVOX™ Oblique Lateral Spinal System (Medtronic, Memphis, USA) plate and bone screw components are used as a new supplemental fixation device for the OLIF procedure. The Pivox 2-hole anterior plate allows for variable angle placement of the screws from 0° to 15° in the cephalic/caudal plane. One concern that should be addressed is whether this construct can effectively improve the stability and reduce cage subsidence. However, related research on lateral plate has been rarely reported.

The aim of this study was to compare the incidence and degree of cage subsidence between stand-alone OLIF and OLIF combined with lateral plate fixation in the treatment of patients with degenerative lumbar spinal diseases.

## Methods

### Patient selection

This was a retrospective study of patients who underwent OLIF with and without lateral fixation from January 2020 to December 2020. A total of 41 patients (47 lumbar spinal levels) were included in the study. 20 patients (21 lumbar spinal levels) underwent stand-alone OLIF and the other 21 patients (26 lumbar spinal levels) underwent OLIF with lateral plate fixation. All procedures were performed by one senior spine surgeon. The study was approved by the ethical committee of the authors’ hospital.

The inclusion criteria were: (1) patients who underwent OLIF with and without lateral plate fixation at L2 to L5 levels;(2) diagnosis of degenerative lumbar disease with symptoms, including spinal stenosis, degenerative spondylolisthesis, discogenic low back pain or adjacent segment disease; (3) failure of conservative treatment over at least three months; (4) and a minimum follow-up of three months. Exclusion criteria were patients with additional posterior fixation, severe spinal stenosis, high-grade degenerative spondylolisthesis, infections, traumas, tumors, isthmic spondylolisthesis, severe osteoporosis, and incomplete medical records.

### Surgical procedure

In the SA-OLIF group, the surgeries were performed by the standard procedure [11]. The patients were placed in the right lateral decubitus position. A skin incision was made 4–10 cm anterior to the center of the marked disc. The retroperitoneal space was reached by blunt dissection of the abdominal muscle. A serial of dilators was placed to establish the anatomical oblique lateral corridor. After the disc was removed and endplates were prepared, the appropriate size of cage (Clydesdale Spinal System, Medtronic, or Oracle system, Synthes) filled with demineralized bone matrix and artificial bone material was inserted in the optimal position.

In the OLIF + LP group, the surgery was performed by the standard procedure [11], as above. After intervertebral cage insertion (PIVOX™ Oblique Lateral Spinal System, Medtronic), the Pivox plate was placed to the lateral vertebral bodies using two screws that locked with the plate at different angles from 0° to 15° in the cephalic/caudal plane. None of the patients in either group underwent direct decompression.

### Demographic and perioperative data collection

Demographic data included gender, age, body mass index (BMI), bone mineral density (BMD), preoperative diagnosis, and follow-up time. BMD was measured by quantitative computed tomography. Perioperative data included the number of operative levels, operative time, estimated intraoperative blood loss (EBL), and complications of intraoperation and postoperation. Operative time and EBL were calculated as the values divided by the number of operative levels.

### Clinical and radiological evaluation

Clinical outcome was evaluated using the visual analog scale (VAS) for back pain and leg pain, the Japanese Orthopaedic Association (JOA) score, and the Oswestry disability index (ODI) for degenerative lumbar diseases at preoperation and last follow-up.

**Fig. 1:**
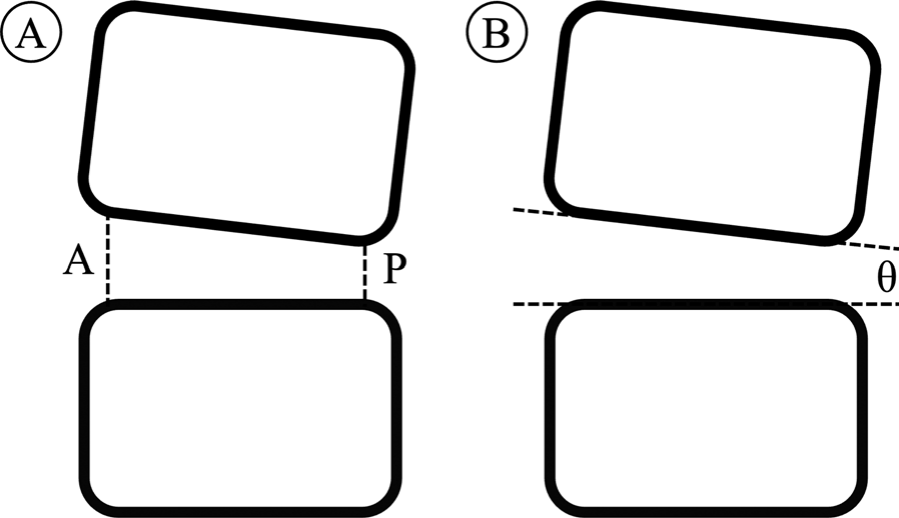
Radiologic measurement. **a** Disc height (DH), DH = (A + P)/2; **b** Segmental lordosis (SL), SL = θ. (A: anterior, P: posterior)

Radiographic evaluation included disc height and segmental lordosis angle based on standing neutral lateral radiographs performed before surgery, at 3 days postoperatively, and the last follow-up (Fig. 1). The disc height (DH) was measured as the average of the anterior and posterior margins of intervertebral height. The segmental lordosis (SL) was measured as the angle between the superior endplate and inferior endplate in the disc space. Cage subsidence was assessed at 3 days postoperatively and the last follow-up using the classification presented by Marchi et al. [8], which based on the amount of cage subsidence into the vertebral endplates in standing neutral lateral radiographs (Grade 0, 0%–24%; Grade I, 25%–49%; Grade II, 50%–74%; and Grade III, 75%–100%).

### Statistical analysis

All statistical analyses were performed using SPSS software version 20.0 (SPSS, Chicago, IL). Results were reported as mean ± standard deviation. The radiographic measurements were performed by two investigators using Surgimap software (version: 2.3.2.1, Nemaris, Inc., New York, NY, USA). The interobserver reliability was evaluated by intra-class correlations coefficients (ICCs), and classified as poor (0–0.39), fair to good (0.4–0.74), or excellent (0.75–1). Independent sample t-test, Paired t-test, Chi-square test, and Fisher exact test were used for comparison between variables. Statistical significance was accepted at *P* < 0.05.

## Results

### Demographic and perioperative data

The demographic data of the patients are shown in Table 1. The mean follow-up time was 6.6 ± 2.9 months in the stand-alone OLIF group and 6.1 ± 1.8 months in the OLIF + LP group. There were no statistical differences in the demographic data.

**Table 1:** Patient demographic data

	SA-OLIF (*n* = 20)	OLIF + LP (*n* = 21)	*p* value
Age, years	61.4 ± 12.0	59.0 ± 9.5	0.489
Gender, female/male	8/12	7/14	0.678
BMI, kg/m^2^	25.2 ± 3.6	25.5 ± 3.1	0.737
BMD, mg/cm^3^	89.2 ± 25.4	96.1 ± 13.0	0.279
*Preop diagnosis, n (%)*			
Degenerative disc disease with spinal stenosis	10 (50.0)	10 (47.6)	0.817
Degenerative spondylolisthesis	6 (30.0)	8 (38.1)	
*Other diseases*	4 (20.0)	3 (14.3)	
Lumbar segmental instability	1 (5.0)	2 (9.5)	-
Discogenic low back pain	2 (10.0)	1 (4.8)	
Adjacent segment disease	1 (5)	0 (0)	
Follow-up, months	6.6 ± 2.9	6.1 ± 1.8	0.453

SA-OLIF: stand-alone oblique lumbar interbody fusion; OLIF + LP: oblique lumbar interbody fusion combined with lateral plate fixation; BMI: body mass index; BMD: bone mineral density

The perioperative data of the patients are shown in Table 2. In the stand-alone OLIF group, a single level was treated in 19 patients, and double levels were treated in 1 patient. In the OLIF + LP group, a single level was treated in 16 patients, double levels were treated in 5 patients. There were no statistical differences between both groups in terms of operation time and EBL. The total perioperative complication rate was 15% (3/20) in the stand-alone OLIF group and 23.1% (6/26) in the OLIF + LP group. Perioperative complications were lumbar plexus injury (*n* = 1) and revision (n = 2) in the stand-alone OLIF group, and lumbar plexus injury (n = 2), sympathetic chain injury (n = 1), pseudohernia (n = 1), and revision (n = 2) in the OLIF + LP group.

**Table 2:** Patient perioperative data

	SA-OLIF (*n* = 20)	OLIF + LP (*n* = 21)	*p* value
*No. of operative levels, n (%)*			
Single	19 (95.0)	16 (76.2)	0.184
Double	1 (5.0)	5 (23.8)	
Operative time per 1-level (min)	120.8 ± 32.8	103.7 ± 23.7	0.064
Estimated blood loss per 1-level (ml)	62 ± 24.0	52.3 ± 36.2	0.325
*Perioperative complications, n (%)*			
Lumbar plexus injury	1 (5.0)	2 (12.5)	-
Sympathetic chain injury	–	1 (6.3)	
Pseudohernia	–	1 (6.3)	
Revision	2 (10.0)	2 (12.5)	

SA-OLIF: stand-alone oblique lumbar interbody fusion; OLIF + LP: oblique lumbar interbody fusion combined with lateral plate fixation

### Clinical outcomes

The clinical data of the patients are shown in Table 3. There were no significantly differences between the groups in the VAS, JOA, and ODI scores at preoperation and the last follow-up. All of the clinical score (Vas, JOA, and ODI) improved in both groups at the last follow-up, compared with the preoperative data (all *P* < 0.05).

**Table 3:** Clinical evaluation

	SA-OLIF (*n* = 20)	OLIF + LP (*n* = 21)	*p* value
*VAS for back pain*			
Preop	3.4 ± 2.1	3.1 ± 2.1	0.615
Last follow-up	1.6 ± 2.3*	1.3 ± 1.3*	0.707
*VAS for leg pain*			
Preop	5.4 ± 2.4	4.9 ± 2.7	0.519
Last follow-up	1.1 ± 2.2*	0.9 ± 1.9*	0.788
*JOA*			
Preop	14.2 ± 3.0	15.7 ± 3.6	0.161
Last follow-up	22.2 ± 6.0*	24.0 ± 4.1*	0.249
*ODI (%)*			
Preop	47.1 ± 7.7	42.5 ± 16.0	0.246
Last follow-up	12.6 ± 14.0*	16.3 ± 14.3*	0.409

SA-OLIF: stand-alone oblique lumbar interbody fusion; OLIF + LP: oblique lumbar interbody fusion combined with lateral plate fixation

^*^Means statistically significant, compared with preoperative data

### Radiological outcomes

**Fig. 2:**
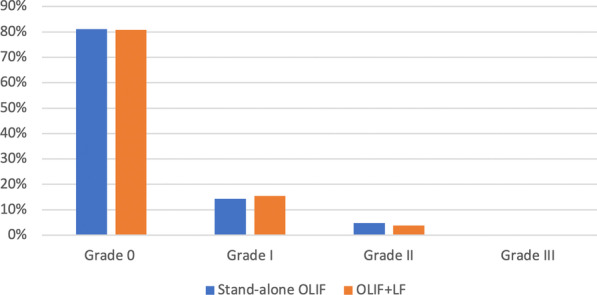
Graph showing the subsidence rate between the groups at follow-up periods. OLIF, oblique lumbar interbody fusion; SA, stand-alone; LP, lateral plate fixation

The radiological data of the patients are shown in Table 4. The interobserver reliabilities for the radiological measurements were excellent (ICCs for DH:0.923, SL:0.871). The cages (average lordosis of 6.6° and average width of 19.2 mm) were often used in the SA-OLIF group, and the cages (average lordosis of 8.9° and average width of 20.5 mm) were often used in the OLIF + LP group. There were no significant differences in the SL or DH between the groups before surgery, at 3 days postoperatively, or the last follow-up. The overall incidence of subsidence at the last follow-up is shown in Fig. 2. More than 50% of cage settlement (grade II or III) is defined as high-grade subsidence [8]. The high-grade subsidence rate was 4.8% (1/21) in the stand-alone OLIF group and 3.8% (1/26) in the OLIF + LP group.

**Table 4:** Radiologic evaluation

	SA-OLIF (21 levels)	OLIF + LP (26 levels)	*p* value
*Fusion levels, n*			
L3/4	3	7	0.475
L4/5	18	19	
*Cage size*			
Cage lordosis	6.6 ± 0.9	8.9 ± 3.0	**0.001**
Cage height	13.6 ± 1.0	13.6 ± 1.6	0.815
Cage length	53.3 ± 2.4	54.0 ± 3.7	0.328
Cage width	19.2 ± 1.9	20.5 ± 1.5	**0.010**
*Disc height*			
Preop	9.4 ± 2.1	9.4 ± 2.4	0.992
Postop	12.8 ± 2.2*	12.6 ± 2.3*	0.775
Last follow-up	10.4 ± 2.8	11.0 ± 3.4*	0.537
*Segmental lordosis*			
Preop	7.3 ± 2.9	6.7 ± 3.6	0.563
Postop	9.4 ± 2.3*	9.5 ± 3.4*	0.900
Last follow-up	8.8 ± 3.4	8.6 ± 3.8	0.951
*Cage subsidence, n (%)*			
Grade 0 (0–24%)	17 (81.0)	21 (80.8)	0.984
Grade I (25–49%)	3 (14.3)	4 (15.4)	
Grade II (50–74%)	1 (4.8)	1 (3.8)	
Grade III (75–100%)	0 (0)	0 (0)	

SA-OLIF: stand-alone oblique lumbar interbody fusion; OLIF + LP: oblique lumbar interbody fusion combined with lateral plate fixation

Bold means statistically significant

^*^Means statistically significant, compared with preoperative data

**Fig. 3:**
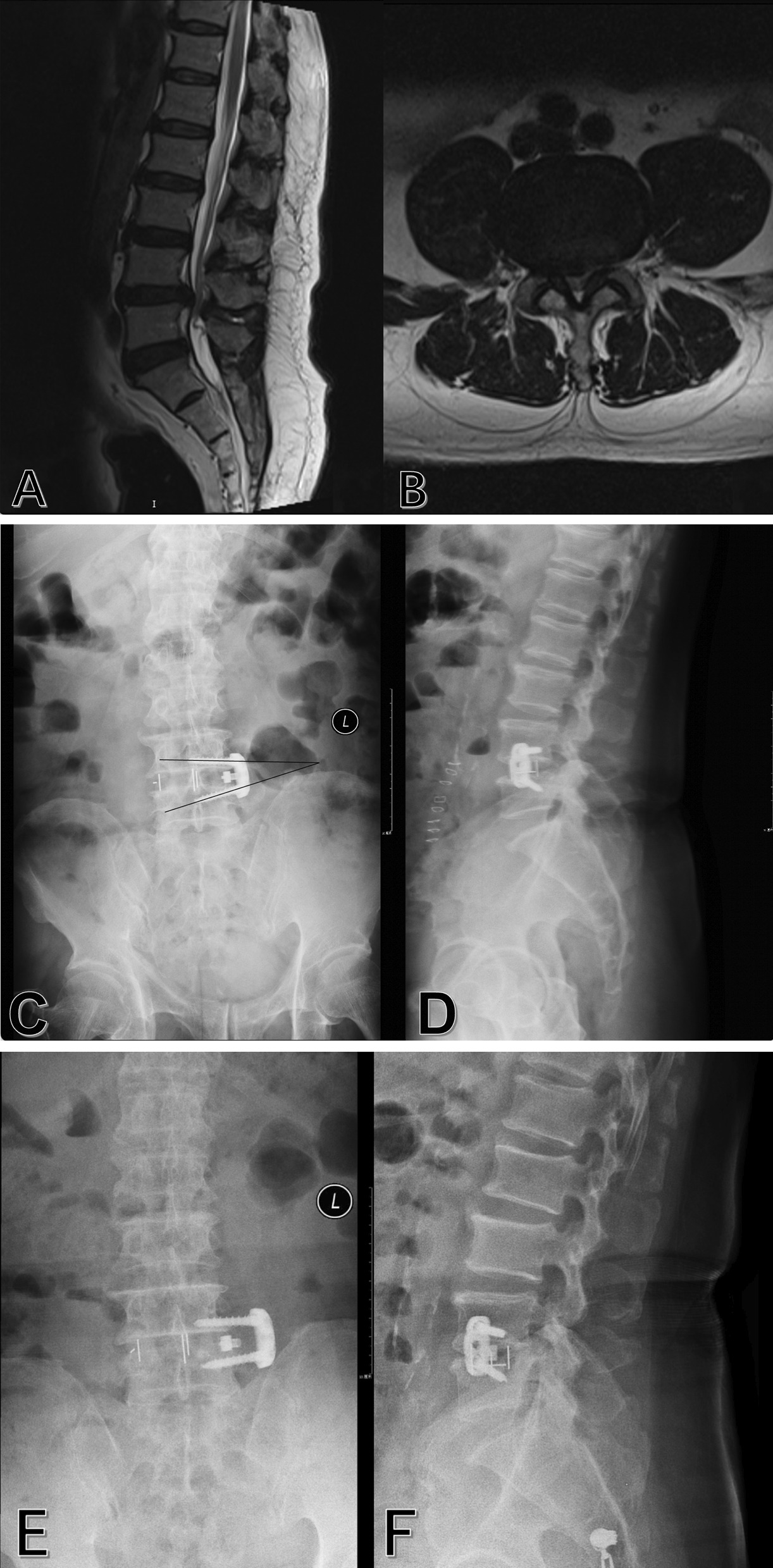

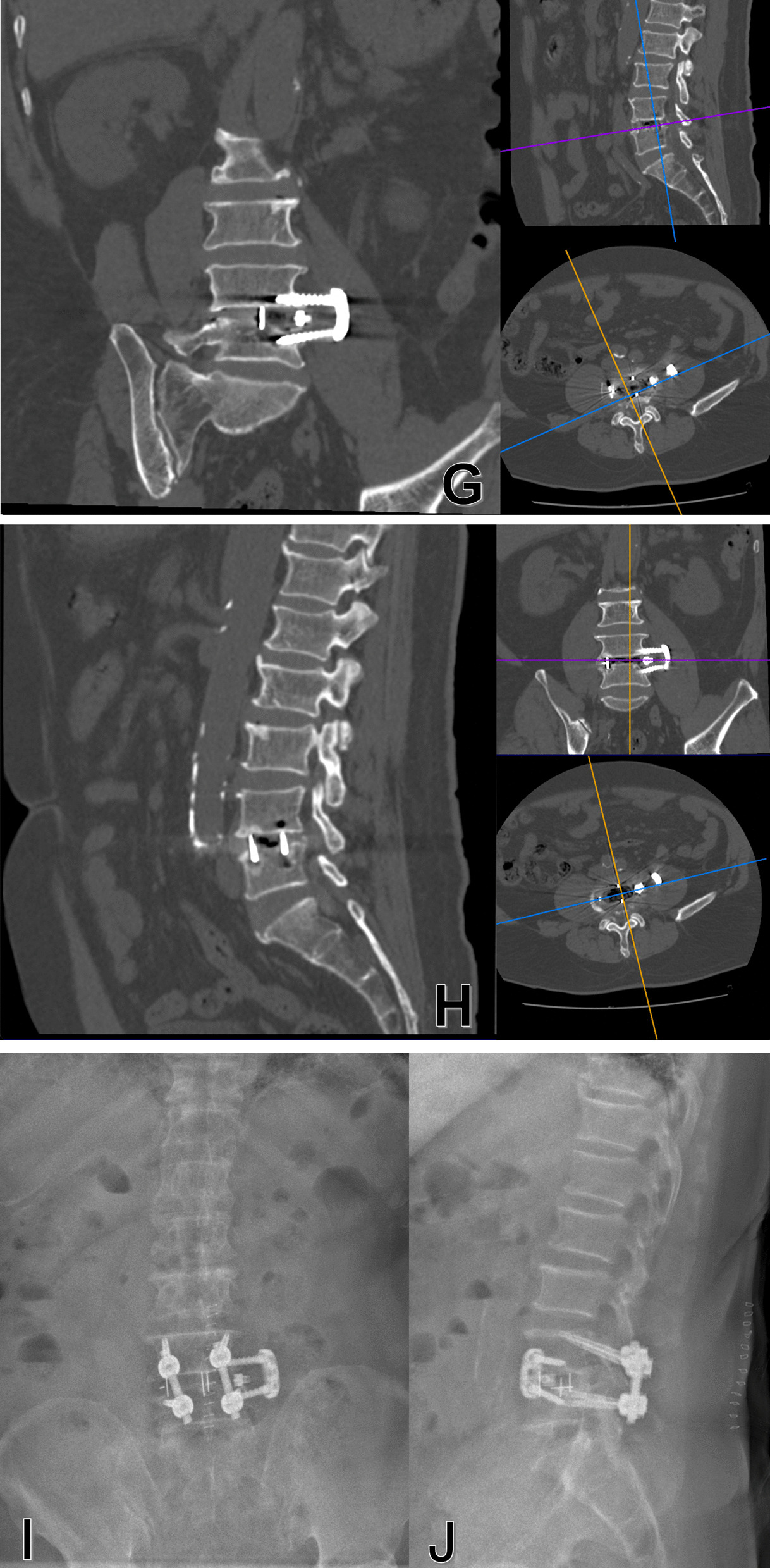
Images obtained in a 77-year-old man who presented with back pain and neurogenic intermittent claudication (BMI: 29.7 kg/m^2^; BMD: 114.1 mg/cm^3^). **a** and **b** Preoperative magnetic resonance imaging revealed moderate stenosis at L_4–5_. **c** and **d** Postoperative anteroposterior and lateral radiographs revealed the satisfactory position of hardware. The angle between screws was 17.8° in the anteroposterior radiograph. **e** and **f** Anteroposterior and lateral radiographs at 7 weeks after the initial surgery revealed cage subsidence and the migration of lateral plate and screws. The angle between screws was 7.9° in the anteroposterior radiograph. **g** Computed tomography coronal reconstruction confirmed the migration of lateral plate and screws. **h** Computed tomography sagittal reconstruction confirmed cage subsidence. **i** and **j** Anteroposterior and lateral radiographs after revision surgery with cortical bone trajectory screw placement in midline lumbar fusion (MIDLF) and left L4 laminotomy

One case involved cage subsidence and migration of lateral plate in the OLIF + LP group. This phenomenon was presented in Fig. 3.

## Discussion

OLIF via a retroperitoneal pre-psoas approach is a safe and effective technique used for lumbar interbody fusion [3, 7, 11]. The reason for utilizing OLIF combined with lateral plate fixation is that adding lateral fixation may biomechanically increase the stability of an interbody fusion construct, reduce cage subsidence and improve fusion rate. But the effect of lateral plate on cage subscidence were seldom reported.

The results of this study showed that additional lateral plate fixation did not have certain effect on cage subsidence. Regarding biomechanics of supplemental lateral plate fixation, lateral plate fixation has been reported to provide more stability in lateral bending compared with stand-alone condition and was not different from stand-alone condition in flexion–extension [9, 10, 12]. It is controversial that supplemental lateral plate fixation can significantly enhance more stability than stand-alone condition in axial rotation [9, 10]. The position of lateral plate is associated with stability of biomechanics that an anteriorly applied plate might better resist this extension-flexion of motion and a laterally applied plate might provide more stable in lateral bending [13]. In fact, lateral plate fixation is considered to be a risk factor for cage subsidence and vertebral fracture, owing to the devastating effect of the screw on the subchondral trabecular support [14]. Chen et al. [6] reported there was not significantly difference about cage subsidence in lateral interbody fusion with and without supplemental fixation. Based on the above literature, the cage subsidence in OLIF + LP group may be caused by the failure of lateral plate fixation to increase stability in flexion–extension and the destructive effect of the screw on the subchondral trabeculae.

Cage subsidence was associated with revision risk but not clinical outcomes. Chio et al. [15] conducted cage subsidence had no relationship with recurrence of symptoms. Conversely, Tempel et al. [16] reported that cage subsidence grade had a strong correlation with the risk of revision surgery following stand-alone LLIF. In their study, the revision rate in patients with high-grade subsidence was more than that in patients with low-grade subsidence which was consistent with our research. In our study, the mean incidence rate of revision in the 9 patients with radiographic evidence of cage subsidence reached 44% (4/9). The causes of revision cases were the failure of indirect decompression and intervertebral stability dueing to high-grade cage subsidence.

**Fig. 4:**
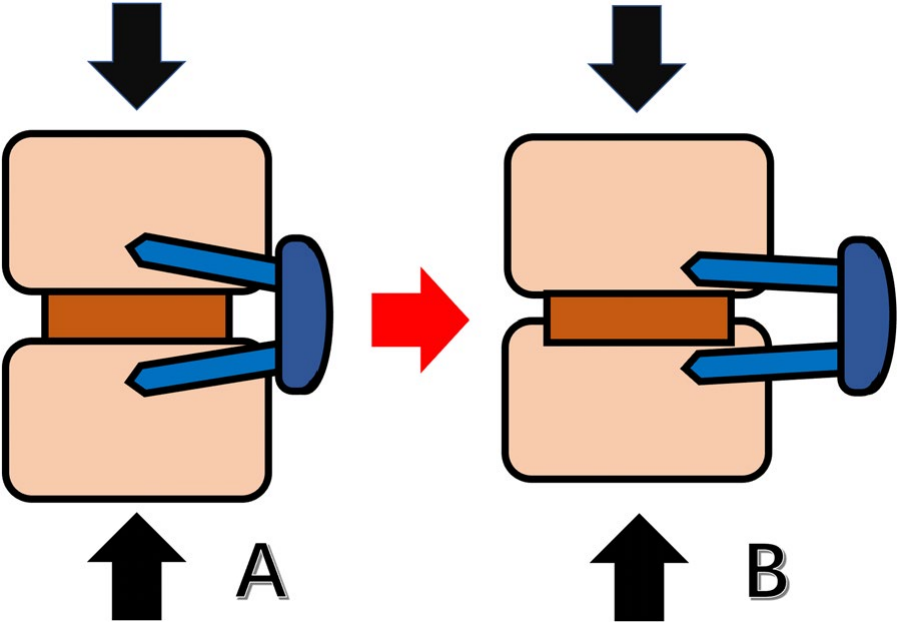
Schematic showing how the lateral plate migration occurred. A, vertical loading of the fusion segment is converted to the horizontal loading of the lateral plate and screws because of the specific angle between the screws and the plate. B, When the cage subsidence occurs, the horizontal loading exceeds the pullout strength of the screws, resulting in the change of angle between screws and the migration of lateral plate and screws

The additional lateral plate fixation did not significantly decrease revision rate, and even may increase the risk of lateral plate failure. Previous studies showed that the incidence of complications associated with lateral plate was 5.9%-15% [17, 18], which was usually associated with vertebral fractures and cage subsidence. However, none of these reported cases were performed with severe lateral plate migration. The Pivox plate has the potential of migration due to its special design that two screws locked with the plate at different angles from 0° to 15° in the cephalic/caudal plane. We speculate one possible mechanism that the progression of cage subsidence contributed to intervertebral height reduction and screw loading increment. The migration of plate and screws might have developed due to the specific angle between the screw and the plate, which caused the increased vertical loading of the fusion segment is converted to the horizontal loading of the lateral plate and screws (Fig. 4).

The results also suggested that the addition of lateral plate fixation did not increase the operation time and estimated intraoperative blood loss significantly. However, the additional internal fixation could increase intraoperative radiation exposure. In this study, the reason for less operation time and estimated intraoperative blood loss per 1-level in the OLIF + LP group was that the number of patients with double fused levels was relatively more in the OLIF + LP group. The clinical score was similar between the stand-alone OLIF and OLIF + LP groups at the postoperative period.

The present study had several important limitations. First, the sample size was small, because our study was the first analysis about the Pivox plate and the result was not ideal. Second, the follow-up period was also relatively short. However, previous studies proven the 6–18 weeks were adequate to access cage subsidence [8, 15, 19–27]. Marchi et al. [8] reported 90.2% of cage subsidence was identified at the 6 weeks postoperatively and there was no incidence of subsidence or worsening of subsidence after 3 months postoperatively. Agarwal et al. [27] reported cage subsidence occurred on average 4.7 months postoperatively. Cheung et al. [21] reported that 81.8% of cage subsidence occurred within the first 3 months after surgery. Choi et al. [15] founded the 4-month actuarial rates for developing cage subsidence were 70.7%. Third, we did not evaluate the fusion rate because fusion had not yet taken place within the first 6 months after surgery without bone morphogenetic proteins.

## Conclusion

The additional lateral plate fixation does not appear to be more effective to prevent cage subsidence in the oblique lumbar interbody fusion, compared with stand-alone technique. If severe cage subsidence occurs, it may result in lateral plate migration in OLIF combined with lateral plate fixation.

## Data Availability

The data used to support the findings of this study are available from the corresponding author upon request.

## Abbreviations

OLIF: Oblique lumbar interbody fusion
SA-OLIF: Stand-alone oblique lumbar interbody fusion
OLIF + LP: Oblique lumbar interbody fusion combined with lateral plate fixation
DH: Disc height
SL: Segmental lordosis
BMI: Body mass index
BMD: Bone mineral density
VAS: Visual analog scale
JOA: Japanese Orthopaedic Association score
ODI: The Oswestry disability index

## References

[1] Mobbs RJ, Phan K, Malham G, Seex K, Rao PJ (2015). Lumbar interbody fusion: techniques, indications and comparison of interbody fusion options including PLIF, TLIF, MI-TLIF, OLIF/ATP, LLIF and ALIF. J Spine Surg.

[2] Bochicchio M, Aicale R, Romeo R, Nardi PV, Maffulli N (2021). Mini-invasive bilateral transfacet screw fixation with reconstruction of the neural arch for lumbar stenosis: a two centre case series. Surgeon.

[3] Li R, Li X, Zhou H, Jiang W (2020). Development and application of oblique lumbar interbody fusion. Orthop Surg.

[4] Sato J, Ohtori S, Orita S, Yamauchi K, Eguchi Y, Ochiai N (2017). Radiographic evaluation of indirect decompression of mini-open anterior retroperitoneal lumbar interbody fusion: oblique lateral interbody fusion for degenerated lumbar spondylolisthesis. Eur Spine J.

[5] Lee KY, Lee JH, Kang KC, Shin SJ, Shin WJ, Im SK (2020). Minimally invasive multilevel lateral lumbar interbody fusion with posterior column osteotomy compared with pedicle subtraction osteotomy for adult spinal deformity. Spine J.

[6] Chen E, Xu J, Yang S, Zhang Q, Yi H, Liang D (2019). Cage subsidence and fusion rate in extreme lateral interbody fusion with and without fixation. World Neurosurg.

[7] Mehren C, Mayer HM, Zandanell C, Siepe CJ, Korge A (2016). The oblique anterolateral approach to the lumbar spine provides access to the lumbar spine with few early complications. Clin Orthop Relat Res.

[8] Marchi L, Abdala N, Oliveira L, Amaral R, Coutinho E, Pimenta L (2013). Radiographic and clinical evaluation of cage subsidence after stand-alone lateral interbody fusion. J Neurosurg Spine.

[9] Reis MT, Reyes PM, Bse, Altun I, Newcomb AG, Singh V et al. Biomechanical evaluation of lateral lumbar interbody fusion with secondary augmentation. J Neurosurg Spine. 2016;25(6):720–6. 10.3171/2016.4.Spine151386. 10.3171/2016.4.SPINE151386 27391398

[10] Fogel GR, Parikh RD, Ryu SI, Turner AW (2014). Biomechanics of lateral lumbar interbody fusion constructs with lateral and posterior plate fixation: laboratory investigation. J Neurosurg Spine.

[11] Woods KR, Billys JB, Hynes RA (2017). Technical description of oblique lateral interbody fusion at L1–L5 (OLIF25) and at L5–S1 (OLIF51) and evaluation of complication and fusion rates. Spine J.

[12] Cappuccino A, Cornwall GB, Turner AW, Fogel GR, Duong HT, Kim KD et al. Biomechanical analysis and review of lateral lumbar fusion constructs. Spine (Phila Pa 1976). 2010;35(26 Suppl):S361–7. 10.1097/BRS.0b013e318202308b. 10.1097/BRS.0b013e318202308b 21160401

[13] Nayak AN, Gutierrez S, Billys JB, Santoni BG, Castellvi AE (2013). Biomechanics of lateral plate and pedicle screw constructs in lumbar spines instrumented at two levels with laterally placed interbody cages. Spine J.

[14] Tender GC (2014). Caudal vertebral body fractures following lateral interbody fusion in nonosteoporotic patients. Ochsner J.

[15] Choi JY, Sung KH (2006). Subsidence after anterior lumbar interbody fusion using paired stand-alone rectangular cages. Eur Spine J.

[16] Tempel ZJ, McDowell MM, Panczykowski DM, Gandhoke GS, Hamilton DK, Okonkwo DO et al. Graft subsidence as a predictor of revision surgery following stand-alone lateral lumbar interbody fusion. J Neurosurg. Spine. 2018;28(1):50–6. 10.3171/2017.5.Spine16427. 10.3171/2017.5.SPINE16427 29125429

[17] Dua K, Kepler CK, Huang RC, Marchenko A (2010). Vertebral body fracture after anterolateral instrumentation and interbody fusion in two osteoporotic patients. Spine J.

[18] Le TV, Smith DA, Greenberg MS, Dakwar E, Baaj AA, Uribe JS (2012). Complications of lateral plating in the minimally invasive lateral transpsoas approach. J Neurosurg Spine.

[19] Beutler WJ, Peppelman WC (2003). Anterior lumbar fusion with paired BAK standard and paired BAK Proximity cages: subsidence incidence, subsidence factors, and clinical outcome. Spine J.

[20] Schiffman M, Brau SA, Henderson R, Gimmestad G (2003). Bilateral implantation of low-profile interbody fusion cages: subsidence, lordosis, and fusion analysis. Spine J.

[21] Cheung KM, Zhang YG, Lu DS, Luk KD, Leong JC. Reduction of disc space distraction after anterior lumbar interbody fusion with autologous iliac crest graft. Spine (Phila Pa 1976). 2003;28(13):1385–9. 10.1097/01.Brs.0000067093.47584.Ca. 10.1097/01.BRS.0000067093.47584.CA 12838095

[22] Kumar A, Kozak JA, Doherty BJ, Dickson JH. Interspace distraction and graft subsidence after anterior lumbar fusion with femoral strut allograft. Spine (Phila Pa 1976).1993; 18(16):2393–2400. 10.1097/00007632-199312000-00005. 10.1097/00007632-199312000-00005 8303439

[23] Lin GX, Kotheeranurak V, Zeng TH, Mahatthanatrakul A, Kim JS (2019). A longitudinal investigation of the endplate cystic lesion effect on oblique lumbar interbody fusion. Clin Neurol Neurosurg.

[24] Malham GM, Parker RM, Blecher CM, Seex KA (2015). Assessment and classification of subsidence after lateral interbody fusion using serial computed tomography. J Neurosurg Spine.

[25] Marchi L, Oliveira L, Amaral R, Castro C, Coutinho T, Coutinho E (2012). Lateral interbody fusion for treatment of discogenic low back pain: minimally invasive surgical techniques. Adv Orthop.

[26] Tokuhashi Y, Ajiro Y, Umezawa N. Subsidence of metal interbody cage after posterior lumbar interbody fusion with pedicle screw fixation. Orthopedics. 2009;32(4). 19388615

[27] Agarwal N, White MD, Zhang X, Alan N, Ozpinar A, Salvetti DJ *et al*. Impact of endplate-implant area mismatch on rates and grades of subsidence following stand-alone lateral lumbar interbody fusion: an analysis of 623 levels. J Neurosurg Spine. 2020;33(1):12–6. 10.3171/2020.1.Spine19776. 10.3171/2020.1.SPINE19776 32114533

